# Optimal Production of Protein Hydrolysates from Monkfish By-Products: Chemical Features and Associated Biological Activities

**DOI:** 10.3390/molecules25184068

**Published:** 2020-09-06

**Authors:** José Antonio Vázquez, Araceli Menduíña, Margarita Nogueira, Ana I. Durán, Noelia Sanz, Jesus Valcarcel

**Affiliations:** 1 Grupo de Biotecnología y Bioprocesos Marinos, Instituto de Investigaciones Marinas (IIM-CSIC), C/Eduardo Cabello, 6, CP 36208 Vigo, Galicia, Spain; araceli@iim.csic.es (A.M.); marga@iim.csic.es (M.N.); anais@iim.csic.es (A.I.D.); nsanz@iim.csic.es (N.S.); jvalcarcel@iim.csic.es (J.V.); 2 Laboratorio de Reciclado y Valorización de Materiales Residuales (REVAL), Instituto de Investigaciones Marinas (IIM-CSIC), Eduardo Cabello, 6, CP 36208 Vigo, Galicia, Spain; 3 Laboratorio de Bioquímica de Alimentos, Instituto de Investigaciones Marinas (IIM-CSIC), Eduardo Cabello, 6, CP 36208 Vigo, Galicia, Spain

**Keywords:** protein recovery, monkfish by-products, fishing waste valorization, fish protein hydrolysates, bioactive, sustainability

## Abstract

The aim of this work was the recovery of protein substrates from monkfish waste (heads and viscera) generated in the on-board processing of this species. Initially, the effect of pH, temperature, and protease concentration was studied on mixtures of a 1:1 ratio (*w/v*) of monkfish heads/water. The optimal conditions of proteolytic digestion were established at 57.4 °C, pH 8.31, [Alcalase] = 0.05% (*v/w*) for 3 h of hydrolysis. Later on, a set of hydrolysis at 5L-pH-stat reactor were run under the aforementioned conditions, confirming the validity of the optimization studies for the head and viscera of monkfish. Regarding the chemical properties of the fish protein hydrolysates (FPH), the yield of digestion was higher than 90% in both cases and the degrees of hydrolysis and the soluble protein content were not especially large (<20% and <45 g/L, respectively). In vitro digestibility was higher than 90% and the percentage of essential amino acids ranged from 40 to 42%. Antioxidant activities were higher in viscera FPH, and antihypertensive ability was superior in head FPH. The values of number average molecular weights (Mn) of monkfish hydrolysates were 600 Da in the viscera and 947 Da in the head. The peptide size distribution, obtained by size-exclusion chromatography, indicated that the largest presence of peptides below 1000 Da and 200 Da was observed in the viscera FPH.

## 1. Introduction

An increasing global population, the effects of climate change, and the loss of productive croplands driven by urbanization, among other factors, pose substantial challenges for food and nutrition security in the following decades, especially for poorer countries [1,2]. Across the world, many communities rely on fish as their main source of proteins and essential nutrients, as well as in terms of providing a fundamental income. However, many fisheries are currently overexploited, and thus fish production must become sustainable in order to satisfy demand in the long run [3].

Among the range of measures proposed for sustainable fish production, valorization of by-catch and processing by-products allows for an increase in resource efficiency and minimization of waste [4,5]. This strategy has demonstrated the possibility of isolating valuable compounds from specific tissues such as chondroitin sulfate from cartilage, chitin and chitosan from squid pen, or collagen and gelatin from the skin [6,7,8,9]. From a more general perspective, most fish waste contains a significant amount of proteinaceus material, and in consequence, processes to recover this fraction present wider applicability.

A successful procedure to reclaim protein from fish consists in hydrolysis, generally by proteolytic enzymes, to release its constituent peptides into a liquid phase [10]. As a starting point, such fish protein hydrolysates (FPH) represent a source of valuable nutrients for animals and humans, and also as peptones for culture media [11]. Beyond nutrition, many studies have related FPH and specific peptides to a range of bioactivities, including antioxidant, antihypertensive, anti-inflammatory, anti-microbial, and anti-diabetic effects [12,13,14]. Most studies on FPH production have focused on heavily fished and farmed species, such as salmon, trout, mackerel, or tuna [15,16,17]. However, few studies have focused on more modest species in terms of capture volume but with remarkable characteristics from the point of view of valorization. Monkfish are toad-looking fish that are commercially caught in the Atlantic and Indian oceans, usually in order to remove their tails, which only account for 33% of whole-body mass [18]. While niche markets exist for livers and cheeks, in commercial processing, the remaining 66% of monkfish body represents waste.

Several recent studies have described the antioxidant properties of FPH of muscle and liver of *Lophius litulon* species [19,20,21]. In monkfish flesh, specific peptides with high antioxidant activities were isolated and suggested as additives to delay lipid peroxidation in foodstuffs [19], as well as being potential hepatoprotective agents [20]. Interesting as these results may seem, they are not particularly relevant from the valorization perspective, since muscle and also livers can be destined for human consumption. In this regard, an early study chemically hydrolyzed sawdust produced during monkfish filleting, but the products were only basically characterized [22]. More recently, FPH obtained by enzymatic hydrolysis of *Lophius vomerinus* heads showed emulsion stabilization, foaming capacity, and fat absorption properties, as well as antioxidant properties, which are useful in the formulation of food products [23]. In this context, the study of the sensory properties and the microbiological content of the hydrolysates is also fundamental for the development of human foods incorporating FPH from fish substrates [24].

In the present study, a more comprehensive valorization strategy incorporates, as enzymatic hydrolysis substrates, both heads and viscera from a monkfish species not previously reported (*Lophius piscatorius*). Initial optimization of the proteolysis of heads provides the conditions to subsequently evaluate hydrolysis kinetics of the viscera at a 5L-pH-stat scale. Determination of molecular weight, peptide size distribution, along with antioxidant and antihypertensive properties, the latter tested for the first time, provide exhaustive characterization of monkfish by-products.

## 2. Results

The percentage of the viscera, including livers, manually separated from monkfish head waste was of 22%, while the rest was considered the real head substrate. The moisture of these materials was equal in both (84%), the organic matter was significantly higher in the viscera (*p* < 0.05), and the ash content was higher in the head (Table 1). A larger content of total lipids was found in the viscera (*p* < 0.05), and the best level of total proteins was observed in the head (*p* < 0.05). After defatting samples, the content in total protein was statistically similar in both substrates (*p* > 0.05).

### 2.1. Optimization of Alcalase Hydrolysis of Head Monkfish

Initially, we studied the joint effect of pH and temperature (T) on enzyme proteolysis of monkfish by employing a rotatable second order rotatable design (Table S1). The results of this plan conducted to the following empirical equations for each response evaluated (maximum hydrolysis degree—*H_m_* in %, yield of substrate digestion—V_dig_ in %, and total soluble protein—Prs in g/L) as a function of the two independent variables studied:

(1) $$H_{m}=16.71+0.67T+1.77pH+1.13TpH-1.99T^{2}-3.29pH^{2}$$

(2) $$V_{\mathrm{dig}}=91.64-2.89TpH-7.75T^{2}-1.87pH^{2}$$

(3) $$\mathrm{Prs}=39.86+1.31T+3.80pH-4.42T^{2}-5.35pH^{2}$$

**Figure 1:**
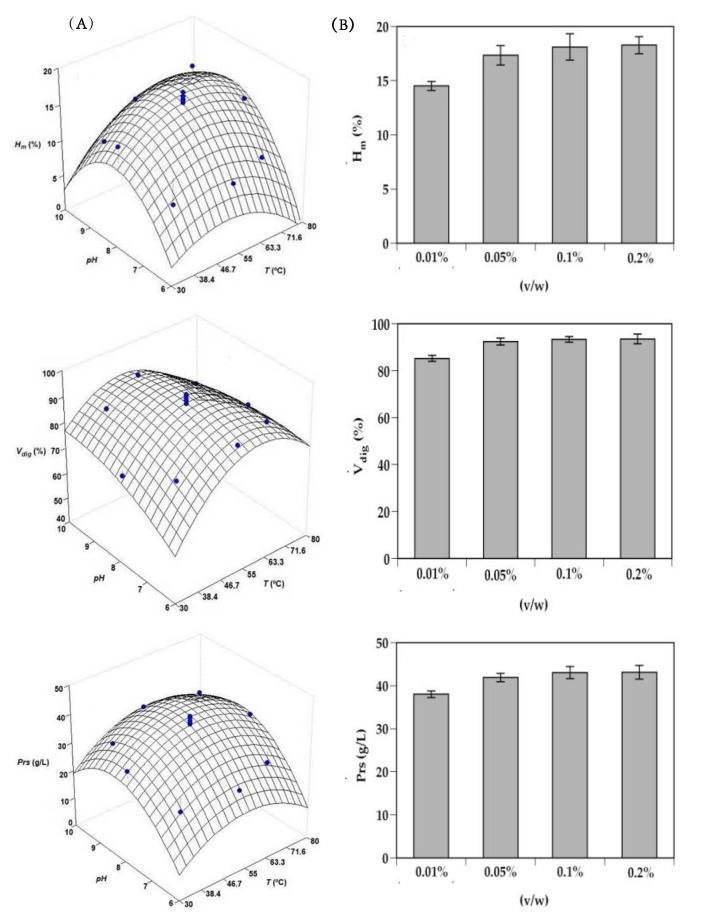
Optimization studies of monkfish head hydrolysis by endogenous Alcalase. Experimental data and predicted response surfaces describing the joint effect of pH and temperature (T) on maximum hydrolysis degree (*H_m_*), yield of substrate digestion (V_dig_), and total soluble protein (Prs) responses (**A**). Individual effect of Alcalase concentration on the same responses (**B**). Error bars are the intervals of confidence for *n* = 2 (replicates of different hydrolysates) and α = 0.05.

The response surfaces predicted by these equations (shown coefficients were all statistically significant, Student’s *t-*test for α = 0.05) are represented in Figure 1A. The degree of correlation between the experimental and simulated data by equations (goodness of fit or degree of explicability of the equations) varied in the range of 0.893–0.934. In turn, the three mathematical models were statistically robust when overcoming Fisher’s *F*-test (α = 0.05) applied to typify the experimental error associated with the experimental data obtained. The optimal values of pH and T that maximize the hydrolysis process were calculated by numerical derivation and were established at 8.31 and 57.4 °C, respectively. Subsequently, and maintaining constant both variables, the effect of protease concentration was also studied (Figure 1B). As can be observed, Alcalase concentration at 0.05%, 0.1%, and 0.2% (*v/w*) showed similar levels of hydrolysis in all the responses analyzed (the differences between them were not statistically significant, *p* > 0.05), but significantly higher than found for the 0.01% level (*p* < 0.05).

### 2.2. Production and Chemical Characteristics of Monkfish FPHs

**Figure 2:**
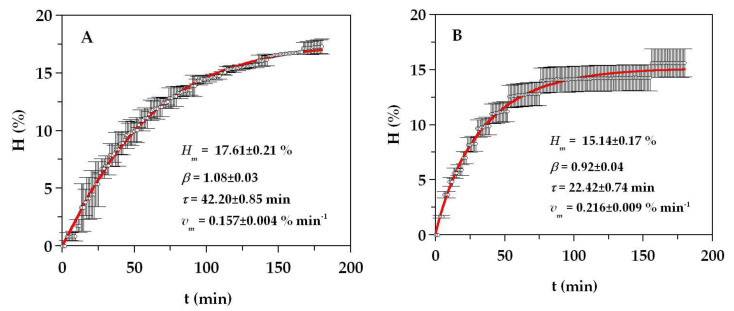
Alcalase hydrolysis of head (**A**) and viscera (**B**) of monkfish waste. Experimental data of hydrolysis degree (symbols) are described by Equation (4) (continuous line). Error bars are the confidence intervals for *n* = 3 (replicates of different hydrolysates) and α = 0.05.

A scaled-up set of hydrolysates from monkfish by-products were produced in a 5L-pH-stat reactor under the optimal conditions found in the previous studies: 0.05% (*v/w*) of Alcalase, pH 8.31, and T of 57.4 °C. For the rest of the conditions, the ratio of solid/liquid (S/L), the continuous agitation, and the time of hydrolysis were established at (1:1), 200 rpm, and 3 h, respectively, in order to reduce operational costs and amount of time taken. Furthermore, at around 3 h, the hydrolysis process was almost completely exhausted, that is, the stationary phase was reached at that time (Figure 2). Similar conditions of agitation, hydrolysis time, and ratio (S/L) were also applied for the production of FPH from salmon and turbot residues [14,16]. The experimental data of hydrolysis degree with clear hyperbolic profiles were simulated by the Weibull equation (4) (Figure 2). The descriptive ability of this equation on the experimental trends was high and almost perfect in both cases (*R^2^* of 0.997 and 0.985 for head and viscera, respectively). The kinetic parameters (*H_m_*, *τ*, *v_m_*, and *β* in Figure 2) were always statistically significant (Student’s *t*-test for α = 0.05), and the consistency of the equation was also confirmed for the two substrates (Fisher’s *F*-test, *p* < 0.0001). The maximum hydrolysis degree (*H_m_*) and the time to achieve the semi-maximum hydrolysis (*τ*) were larger in the hydrolysate of the head, whereas the maximum rate of hydrolysis (*v_m_*) was higher in the viscera.

The non-hydrolysate fraction, essentially the bones, was only recovered in treating the head substrate (around 7%, *w/w* of the initial substrate), and no inorganic fraction was released from the viscera (Table 2). In the viscera, no significant remains of small fish in gastric digestion were found that contributed bones to the production process. No oil was extracted by centrifugation of monkfish hydrolysates due to the presence of oils as emulsions in FPH phases, for which centrifugation was incapable of breaking. The yields of digestion showed by Alcalase on monkfish wastes were remarkable and similar in both cases (>90%). The content of total sugars on liquid hydrolysates was in general low (<1.5 g/L). The level of protein (soluble and total) in the FPHs was found to be comparatively greater through using the head instead of the viscera (*p* < 0.05). In vitro digestibility was always superior to 90% and was statistically indistinguishable between the hydrolysates.

Regarding the percentage composition of amino acids in FPH (Table 3), glutamic acid, glycine aspartic acid, and alanine were the predominant forms. Although for the most part, no significant differences were observed, aspartic acid, alanine, and isoleucine were statistically larger in viscera FPH, and the presence of valine, leucine, and phenylalanine was higher in the hydrolysate of the heads of monkfish. The ratio of essential amino acids per total amino acids was statistically similar in the head (42.1%) and in the viscera (40.3%), with these values being promising for potential human nutritive application of the two hydrolysates.

**Figure 3:**
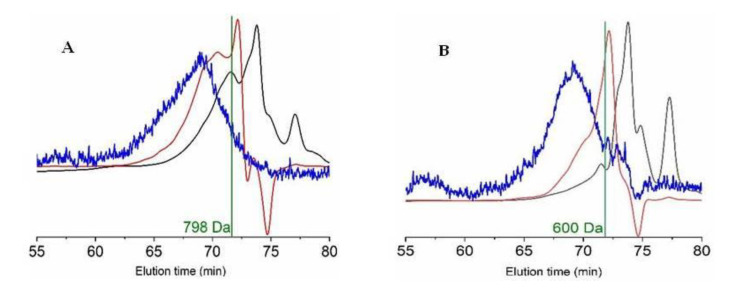

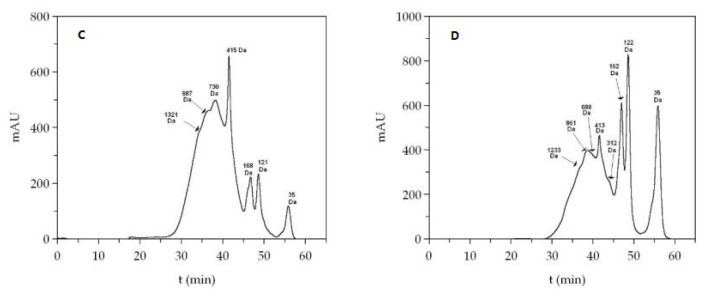
Top: gel permeation chromatography (GPC) eluograms of monkfish hydrolysates from the head (**A**) and viscera (**B**). Red line: refractive index; black line: UV (280 nm); blue line: right angle light scattering; vertical line: number average molecular weight (Mn). Bottom: Size exclusion chromatographic profiles of the head (**C**) and viscera (**D**) hydrolysates.

On the other hand, combining the two chromatographic methods indicated in the Materials and Methods section, the molecular weight and the distribution of peptide sizes for both FPH were determined and are listed in Table 4. The graphical representation of average molecular weights and the profiles of small peptides are also displayed in Figure 3. The most repeated peptide sizes for each FPH (expressed as the number average molecular weight, Mn) were 600 Da and 798 Da for viscera and head hydrolysates, respectively. Another parameter evaluated, the average molecular weight (Mw), showed a larger value in the case of heads (1576 Da) than those found in the viscera (947 Da). The index of polydispersity (PDI) was also greater in head samples than in viscera samples. In concordance with these outcomes, the content of peptides above 1 kDa in head hydrolysates was larger (45%) than those present in viscera hydrolysates (26%). In the same way, the highest fraction of peptides ranging from 0 to 200 Da was found in the hydrolysates of the viscera (35%).

In the dry samples of the hydrolysates, dehydrated by lyophilization, the levels of organic matter were above 70%, with moisture of around 5–9% and with an ash content higher than 18% (Table 5). The percentage of presented fat (as total lipids) was less than 5% in both hydrolysates, and the presence of total was around 70%. In the case of Mo, Lip, and Pr-tN, the differences were statistically significant between the head and viscera FPH. The appearance of the dry FPHs was a small powder that was whitish light brown and with an intense fishy odor. The final yield of the dry powder in relation to the initial substrate reached 6% in monkfish head (60 g of dry FPH per kilogram of head) and 5% in monkfish viscera (50 g of dry FPH per kilogram of viscera). In addition, the content of heavy metals in dry hydrolysates of the head was of 0.48 ± 0.02 ppm, 0.54 ± 0.02 ppm, and <0.10 ppm for Cd, Hg, and Pb, respectively. The concentration of these toxic elements in viscera FPH was 35.7 ± 0.03 ppm for Cd, 0.31 ± 0.03 ppm for Hg, and <0.07 ppm for Pb.

### 2.3. Bioactivities of Monkfish FPH

Finally, samples of monkfish hydrolysates were evaluated in terms of in vitro antioxidant (1,1-diphenyl-2-picrylhydrazyl (DPPH) and 2,2′-azinobis-(3-ethyl-benzothiazoline-6-sulphonic acid (ABTS) scavenging abilities and crocin bleaching effect) and antihypertensive (angiotensin I-converting enzyme (ACE) inhibitory capacity) activities (Table 6). The hydrolysates of viscera and head showed a similar numerical percentage of DPPH, but in the case of ABTS and crocin protocols, the differences between samples were statistically significant (*p* < 0.05), revealing slightly higher antioxidant capacity of viscera hydrolysates. On the contrary, the antihypertensive capacity was statistically higher in head FPH (*p* < 0.05); the inhibition data (as *I_ACE_*) was 64% and 57% for head and viscera hydrolysates, respectively. Regarding the data of half maximal inhibitory concentration (*IC_50_*), the potency of head FPH (931 μg/mL) was somewhat larger than those observed in the viscera FPH (1143 μg/mL).

## 3. Discussion

Monkfish is one of the most valuable commercial species in European markets, but only at around 30–35%; essentially, only the tails are used for human food. Although monkfish head is a tasteful substrate for the preparation of fish fumet, its on-board processing routinely leads to the sea dumping of this material in many fisheries. Therefore, the valorization of monkfish wastes by means of a hydrolysis treatment may be executed in order to recover this protein source, which should be demanded of the fishing industry to comply with the objectives of the circular bioeconomy. The proximate composition of our head monkfish was very similar to that reported for *L. vomerinus* [23]: 86% moisture, 2.6% crude fat, 13.1% organic matter, and 2.6% ash. Moreover, the content of bones in our monkfish species (6.8% *w/w*) was significantly lower than that recovered from the hydrolysis of heads of red scorpionfish (13.2%), blue whiting (13.8%), mackerel (18.3%), grenadier (21.6%), trout (10%), salmon (11%), and turbot (17%) [14,16,25,26].

The selection of a commercial endoprotease, Alcalase, was motivated by its remarkable hydrolytic ability and versatility to digest and liquefy aquaculture wastes [14,16,27], fish discards [11,25,26,28,29], and fish canning by-products [30]. It was also an adequate biocatalyst in the first step in terms of the optimal purification of biological polymers such as glycosaminoglycans [6,8], collagen and derivatives [31], and chitin/chitosan [9,32,33]. In addition, it is an enzyme that is qualified as food grade, that is easy to add, has convenient storage in refrigeration at 4 °C, is fully soluble in aqueous solution, and is reasonably priced for use on an industrial scale. In the present work, the capacity of Alcalase to process monkfish substrates was evidenced, yielding a total digestion of the organic fraction of these wastes, as well as an acceptable maximum degree of hydrolysis (15–17%). Similar values of hydrolysis were also obtained using the muscle of *L. litulon* treated with Trypsin [19,20] and heads of *L. vomerinus* catalyzed with Alcalase [23]. Nevertheless, in the latter case, the degree of hydrolysis was achieved at a shorter time (13–16 min) in comparison with our proposal (3 h), but with incomplete digestion of the heads. In addition, the levels of *H_m_* reported here are in line with others found in the hydrolysis of skins and heads from discarded fish [25,26], but much lower than those observed for FPH from salmon, trout, and turbot by-products [14,16].

Regarding the optimal conditions for the proteolysis of fish materials by Alcalase, the best values of pH and T for monkfish head (57.4 °C, pH 8.31, 0.05% (*v/w*) of enzyme and 3 h of hydrolysis) were in agreement with the salmon head outcomes (56.2 °C/pH 8.27/0.1%/3 h) and lower than established for heads of blue whiting (60 °C/pH 8.65/1%/4 h), turbot (60.3 °C/pH 8.82/0.2%/3 h), and trout (64.2 °C/pH 8.98/0.2%/3 h) [11,14,16]. In a recent work using the head of *L. vomerinus*, the process of digestion was optimized at 62 °C and pH 8.2 for less than 20 min; however, no data of Alcalase concentration was indicated [23]. The content of soluble protein in monkfish FPH were inferior to the levels presented in other fish hydrolysates, but the in vitro digestibilities (greater than 90%) and the composition in amino acids, including essential ones for humans, were similar [14,16,25,26]. On the basis of these highlights, the FPH from the head and viscera of monkfish could be a potential source of protein-rich substrates for the formulation of animal feed and human food supplements [34,35,36,37].

The molecular weights of the protein material in viscera FPH (in terms of Mn and Mw) were smaller than 1 kDa and lower than those obtained for monkfish head (800–1600 Da). Both hydrolysates were also smaller than those employing aquaculture residues (frames and heads) of salmonids (920–1900 Da) [16] and in the same interval of sizes as those of turbot (800–1600 Da) [14]. In the case of the hydrolysate of turbot viscera, the values of Mw were twice as superior as monkfish viscera [14]. The distribution of peptide sizes of monkfish in correspondence with these data of Mw and Mn revealed greater percentages of peptides <1000 Da and <200 Da in the viscera. However, it was not to the level of what may be expected from the value of *H_m_*, as a higher maximum hydrolysis degree was found in the head nor in the proteolysis of viscera wastes. It could be attributed to the fact that endogenous proteases present in monkfish viscera were not inactivated in order to reduce the cost of operation. Moreover, crude material from the viscera could also contain proteins of smaller size than head substrates.

The values of in vitro antioxidants of monkfish hydrolysates were less active and valuable than compared with the data of the hydrolysates from herring, hoki, and trout [38,39,40], but showed similar values of DPPH (around 50%) than those recently reported for the head of *L. vomerinus* [23]. The present bioactivities are also in line with the results reported in FPH generated from discarded fish and turbot by-products [14,41,42,43]. Identical findings of DPPH inhibition percentage were defined for collagen derivatives obtained from the enzyme digestion of fin and skin of salmon [44,45]. On the other hand, the antihypertensive inhibitions (*I_ACE_*) of FPH were always larger than 50% and in concordance with other fish hydrolysates [46,47,48]. No direct comparison with monkfish substrates can be made using literature results, since this is the first report dealing with the production of antihypertensive activities from monkfish FPH. The data of *IC_50_* were in agreement with the previous activities of digested frames of salmon, trout, and turbot [14,16], but they are inferior to the higher activity (lower value of *IC_50_*) of many hydrolysates and specific peptides recovered from fish tissues of tuna, cod, or tilapia [48,49,50]. We observed that viscera monkfish hydrolysates (with Mw<1000 Da) were more active in antioxidant bioassays, whereas the head hydrolysates led, on the contrary, to stronger antihypertensive capacity with bigger protein/peptide size (Mw ≈ 1600 Da). These differences may be supported by the outcomes of other authors that indicated the presence of high antioxidant activity in monkfish peptides of 600–800 Da [19,20], as well as the best antihypertensive ability in fish peptides of 1300–1800 Da [48,49,50].

Finally, the presence of toxic elements in our dry FPH from heads was lower than those quantified for raw heads of *L. vomerinus* (0.95 ppm, 0.40 ppm, and 0.92 ppm for Hg, Pb, and Cd, respectively) [23], and inferior to the limits established by the European community regulation (ECR) 466/2001. However, the level of Cd in viscera FPH (36 ppm) was higher than the regulated value, limiting its use in human formulations.

## 4. Materials and Methods

### 4.1. Monkfish Wastes and Proteolysis Optimization

Heads of monkfish (*Lophius piscatorius*), generated from on-board processing, were kindly supplied by Grupo Pereira S.A. (Vigo, Spain). These substrates were immediately frozen at −18 °C until use. Before hydrolysis, viscera were manually separated from heads, and both were individually ground in a meat mincer and kept again at −18 °C (Figure S1).

The joint effect of pH and temperature (T) on head monkfish hydrolysis were evaluated by a two-variable factorial design (Table S1), using Alcalase 2.4L (2.4 AnsonUnit/g, AU/g enzyme, Nordisk, Bagsvaerd, Denmark) as exogenous protease. The dependent variables (Y) measured were the concentration of soluble protein (Prs), the maximum hydrolysis (*H_m_*), and the yield of digestion (V_dig_). Orthogonal least-squares calculation on factorial design data was used to obtain empirical equations that describe the different responses assessed (Y) in function of T and pH. Student′s *t*-test (α = 0.05) was employed to determine the statistical significance of coefficients. Coefficient of determination (*R*^2^) and adjusted coefficients of determination ($R_{adj}^{2}$) were calculated to establish goodness-of-fit, and Fisher’s *F*-test (α = 0.05) was determined to formalize model consistency. A pH-stat system equipped with a 100 mL enzyme reactor, with control of T and continuous agitation, was used to run these optimization experiments for 3 h, maintaining constant the enzyme concentration (0.2%, *v/w*) and the S/L ratio (1:1). Subsequently, an experiment was launched to study the best protease concentration, maintaining the temperature and the pH in the conditions obtained in the previous factorial plan. In all cases, at the end of hydrolysis, the content from mini reactors were centrifuged (15,000× *g*/20 min), the sediments and supernatants were quantified, and hydrolysates were quickly heated (90 °C/15 min) for enzyme inactivation.

### 4.2. Production of Monkfish Hydrolysates

Monkfish wastes were hydrolyzed at a higher scale (three independent batches for each substrate) using a controlled pH-stat system with a 5 L glass reactor. For both substrates, heads and viscera, the conditions of proteolysis were S/L = 1:1 (2 kg of milled wastes plus 2 L of distilled water), temperature of 57.4 °C, pH = 8.31, [Alcalase] = 0.05% (*v/w*), 200 rpm of agitation, and 3 h of processing. The hydrolysates were filtered (100 μm) to remove non-hydrolyzed material, the hydrolysates were centrifuged (15,000× *g*/20 min) to try to recover oils, and protease deactivation was achieved through the heating (90 °C/15 min) of FPHs (Figure S1). The solid hydrolysates were obtained by freeze-drying. The time-course of hydrolysis was recorded by means of the degree of hydrolysis (*H*, as %) using the pH-stat method [51] and modelled by the following Weibull equation [25,26]:

(4) $$H=H_{m}\left\{1-\exp\left[-\ln2{\left(\frac{t}{\tau }\right)}^{\beta }\right]\right\}\;\mathrm{with}\;v_{m}=\frac{\beta H_{m}\ln2}{2\tau }$$

where *H* is the degree of hydrolysis (%), *t* is the time of hydrolysis (min), *H_m_* is the maximum degree of hydrolysis (%), *β* is a parameter related with the maximum slope of muscle hydrolysis (dimensionless), *v_m_* is the maximum rate of hydrolysis (% min^−1^), and *τ* is the time required to achieve the semi-maximum degree of hydrolysis (min). The yields of digestion of monkfish wastes (V_dig_, % *v/w*) were also quantified as the volume of final FPH produced after bones and enzyme deactivation per the sum in weight of the solid raw material, initial water, and alkalis added for hydrolysis process [25].

### 4.3. Analytical and Biological Determinations

The analytical determinations employed for the chemical characterization of the raw substrates and dried FPHs were (a) the ash, organic matter, and water content [52]; (b) the total protein quantified as total nitrogen × 6.25 [52]; and (c) the total lipids [53]. In the case of the liquid FPHs, we determined (d) the total soluble protein [54], (e) the total sugars [55] in the liquid hydrolysates, (f) the amino acid concentration through employing an amino acid analyzer [56], (g) the in vitro digestibility [57], and (h) the heavy metals using inductively coupled plasma mass spectrometry (ICP-MS) [58]. In addition, the in vitro antihypertensive [59] and various antioxidant (AO) activities were also quantified. Crocin bleaching assay, 1,1-diphenyl-2-picrylhydrazyl (DPPH) method, and ABTS (2,2′-azinobis-(3-ethyl-benzothiazoline-6-sulphonic acid) bleaching protocol were the AO activities determined [60,61]. The molecular weight and the peptide size distribution of FPHs was determined by gel permeation chromatography (GPC-HPLC), as was extensively described in a previous report [14]. Briefly, the GPC system (Agilent 1260 HPLC, equipped with four protein-specific columns: Proteema precolumn, Proteema 30Å, Proteema 100Å, and Proteema 1000Å from PSS (Germany)) and multiple detectors (refractive index, diode array, and dual-angle static light scattering) were used for the calculation of Mw and Mn. Mobile phase was 0.15M ammonium acetate/0.2M acetic acid buffer at pH 4.5 and flow rate = 1 mL/min. Detectors were calibrated with a polyethylene oxide standard (PSS, Germany) of 106 kDa (Mw) and polydispersity index 1.05. Absolute molecular weights were estimated with refractive index increments (dn/dc) of 0.185. For peptide size distribution (<1–2 kDa), we processed the samples of FPH by Agilent 1200 HPLC (UV-detection at 220 nm) using a Superdex peptide 10/300 GL column (GE Healthcare Life Sciences, UK) with 0.1% trifluoroacetic acid in 30% of acetonitrile as mobile phase (flow rate of 0.4 mL/min). The standards used were blue dextran (2 MDa), cytochrome c (12.4 kDa), aprotinin (6.5 kDa), angiotensin II (1046 Da), leucine encephalin (555 Da), Val-Tyr-Val (379 Da), and Gly-Gln (221 Da).

### 4.4. Numerical and Statistical Analyses

Data-fitting procedures and parametric estimations were conducted by minimization of the sum of quadratic differences between observed and model-predicted values, using the non-linear least-squares (quasi-Newton) method provided by the macro “Solver” of the Excel spreadsheet (Microsoft Corporation, Redmond, Washington, DC, USA). Confidence intervals from the parametric estimates (Student’s *t*-test) and consistency of mathematical models (Fisher’s *F*-test) were evaluated by “SolverAid” macro. The significance of comparisons between samples was analyzed by ANOVA, with a significance level of *p* < 0.05.

## 5. Conclusions

In this report, the valorization of wastes generated from monkfish on-board processing (heads and viscera), including the optimization process and bioactive production, was explored. The enzyme (Alcalase) proteolysis of the head was initially optimized to yield complete waste digestion/liquefaction and to produce FPH with an interesting degree of hydrolysis, high in vitro digestibility, relevant content of protein, and remarkable percentage of essential amino acids. Similar highlights were found in the case of viscera hydrolysates. Antioxidant and antihypertensive activities were also determined, indicating acceptable bioactive properties of monkfish FPH in concordance with other fish hydrolysates. Thus, monkfish hydrolysates might be a valuable protein-rich ingredient for the preparation of products for animal and human consumption. Further studies should be conducted to test this assumption, including sensory and microbiological issues of FPH.

## Figures and Tables

**Table 1:** Proximate composition of monkfish by-products in terms of moisture (Mo), organic matter (OM), and ashes (Ash). Total lipids (Lip), proteins (Pr-tN, as total nitrogen x 6.25), and proteins after degreasing samples (Pr-tN) were determined using dried substrates. Error are the confidence intervals for *n* = 3 (independent samples) and α = 0.05. Different letters (a, b) in each column means significant difference between wastes (*p* < 0.05).

WASTE	Mo (%)	OM (%)	Ash (%)	Lip (%)	Pr-tN (%)	Pr-tN (%)
**Head**	84.6 ± 0.9 ^a^	11.5 ± 0.9 ^a^	3.9 ± 1.5 ^a^	3.0 ± 0.9 ^a^	88.3 ± 1.1 ^a^	89.5 ± 11.7 ^a^
**Viscera**	84.6 ± 0.4 ^a^	13.8 ± 0.4 ^b^	1.6 ± 0.1 ^b^	8.7 ± 0.4 ^b^	83.0 ± 4.1 ^b^	85.0 ± 4.0 ^a^

**Table 2:** Mass balances and proximal analysis of the products recovered from Alcalase hydrolysates of monkfish by-products. Shown errors are the confidence intervals for *n* = 3 (replicates of different hydrolysates) and α = 0.05. m_b_: percentage of the bones recovered (*w/w* of crude substrate); V_dig_: yield of digestion process; Prs: total soluble protein determined by Lowry method; Pr-tN: total protein determined as total nitrogen x 6.25; TS: total sugars; Dig: digestibility. Different letters (a,b) in each column mean significant difference between fish protein hydroysates (FPH) (*p* < 0.05).

FPH	m_b_ (%)	V_dig_ (%)	Prs (g/L)	Pr-tN (g/L)	TS (g/L)	Dig (%)
**Head**	6.8 ± 4.1	91.3 ± 1.5 ^a^	41.4 ± 1.0 ^a^	43.1 ± 1.1 ^a^	0.89 ± 0.04 ^a^	90.9 ± 0.5 ^a^
**Viscera**	-	90.0 ± 1.9 ^a^	38.0 ± 1.2 ^b^	39.5 ± 1.3 ^b^	1.27 ± 0.12 ^b^	91.8 ± 0.9 ^a^

**Table 3:** Amino acid content of FPH (% or g/100 g total amino acids) from monkfish by-products. OHPro: hydroxyproline; Pr: protein concentration (calculated in g/L) as the total sum of amino acids present in FPH; TEAA/TAA: ratio total essential amino acids for human/total amino acids. Errors are the confidence intervals for *n* = 3 (replicates of independent hydrolysates) and α = 0.05. Different letters (a,b) in each row/amino acid means significant difference between FPH (*p* < 0.05).

Amino Acids	Head	Viscera	Amino Acids	Head	Viscera
**Asp**	9.43 ± 0.27 ^a^	9.97 ± 0.01 ^b^	**Leu**	6.15 ± 0.05 ^a^	5.55 ± 0.13 ^b^
**Thr**	4.03 ± 0.07 ^a^	3.73 ± 0.28 ^a^	**Tyr**	3.56 ± 0.29 ^a^	3.44 ± 0.89 ^a^
**Ser**	5.69 ± 0.09 ^a^	5.56 ± 0.08 ^a^	**Phe**	4.59 ± 0.16 ^a^	4.24 ± 0.10 ^b^
**Glu**	13.59 ± 0.52 ^a^	14.48 ± 1.00 ^a^	**His**	1.92 ± 0.32 ^a^	1.86 ± 0.11 ^a^
**Gly**	11.66 ± 0.60 ^a^	12.19 ± 0.34 ^a^	**Lys**	6.28 ± 0.10 ^a^	5.82 ± 0.40 ^a^
**Ala**	7.28 ± 0.25 ^a^	7.61 ± 0.00 ^b^	**Arg**	6.06 ± 0.09 ^a^	6.13 ± 0.63 ^a^
**Cys**	1.13 ± 0.28 ^a^	1.17 ± 0.03 ^a^	**OHPro**	3.02 ± 0.17 ^a^	2.87 ± 0.15 ^a^
**Val**	3.65 ± 0.09 ^a^	3.53 ± 0.00 ^b^	**Pro**	6.23 ± 0.10 ^a^	5.91 ± 0.57 ^a^
**Met**	3.20 ± 0.22 ^a^	3.12 ± 0.26 ^a^	**TEAA/TAA (%)**	42.06 ± 1.19 ^a^	40.32 ± 1.27 ^a^
**Ile**	2.53 ± 0.21 ^a^	2.80 ± 0.03 ^b^			

**Table 4:** Average molecular weights (as Mn and Mw) of monkfish FPH. Percentage (%) of peptide distribution between molecular weight ranges was also determined. PDI: polydispersity index. The data are presented as mean ± standard deviation. Different letters (a,b) in each column means significant difference between FPH (*p* < 0.05).

FPH	Mn (Da)	Mw (Da)	PDI	0–0.2 kDa	0.2–0.5 kDa	0.5–1 kDa	1–3 kDa	>3 kDa
**Head**	798 ± 91 ^a^	1576 ± 72 ^a^	1.975	11.2 ± 4.0 ^a^	15.8 ± 6.7 ^a^	27.6 ± 3.7 ^a^	35.1 ± 4.4 ^a^	10.3 ± 2.0 ^a^
**Viscera**	600 ± 66 ^b^	947 ± 44 ^b^	1.578	35.1 ± 12.3 ^b^	16.5 ± 5.8 ^a^	22.1 ± 2.9 ^a^	22.8 ± 2.9 ^b^	3.5 ± 0.7 ^b^

**Table 5:** Proximal composition of monkfish dry hydrolysates: moisture (Mo), organic matter (OM), ashes (Ash), total lipids (Lip), and proteins (Pr-tN, as total nitrogen x 6.25). Errors are the confidence intervals for *n* = 3 (independent analysis) and α = 0.05. Different letters (a,b) in each column means significant difference between FPH (*p* < 0.05).

FPH	Mo (%)	OM (%)	Ash (%)	Lip (%)	Pr-tN (%)
**Head**	9.25 ± 0.35 ^a^	72.28 ± 0.34 ^a^	18.47 ± 0.07 ^a^	2.39 ± 0.21 ^a^	69.80 ± 0.64 ^a^
**Viscera**	5.19 ± 0.32 ^b^	75.07 ± 3.91 ^a^	19.74 ± 3.62 ^a^	4.82 ± 0.15 ^b^	67.41 ± 0.13 ^b^

**Table 6:** Bioactivities of FPH from monkfish wastes. Errors shown are the confidence intervals for *n* = 3 (samples from independent hydrolysates) and α = 0.05. *I_ACE_*: inhibitory activity of angiotensin I-converting enzyme (ACE), and *IC*_50_: half maximal inhibitory concentration. Different letters (a,b) in each column means significant difference between FPH (*p* < 0.05).

Heading	ANTIOXIDANT			ANTIHYPERTENSIVE	
FPH	DPPH (%)	ABTS (μg BHT/mL)	Crocin (μg Trolox/mL)	*I_ACE_* (%)	*IC*_50_ (μg protein/mL)
**Head**	45.05 ± 3.25 ^a^	13.52 ± 0.39 ^a^	8.52 ± 0.65 ^a^	63.5 ± 4.2 ^a^	931.3 ± 85.2 ^a^
**Viscera**	49.66 ± 2.65 ^a^	14.47 ± 0.44 ^b^	9.49 ± 0.28 ^b^	56.6 ± 2.3 ^b^	1142.5 ± 97.1 ^b^

## Supplementary Materials

The following are available online, Figure S1: Pictures of the monkfish by-products (raw materials) and hydrolysates. Table S1: Experimental design for the optimization of the FPH.
