# Supplementary material for: Optimal Production of Protein Hydrolysates from Monkfish By-Products: Chemical Features and Associated Biological Activities
Source: Molecules. 2020 Sep 6;25(18):4068. doi: 10.3390/molecules25184068 (PMC7570475; doi:10.3390/molecules25184068)
Supplement: Supplementary file 1 [file molecules-25-04068-s001.pdf]

molecules

MDPI

Article

Optimal Production of Protein Hydrolysates of By-products of Monkfish, Chemical Features and Associated Biological Activities.

José Antonio Vázquez [1,2,*], Araceli Menduíña [1,2], Margarita Nogueira [1,2], Ana I. Durán [1,2], Noelia Sanz [1,3], and Jesus Valcarcel [1,2]

[1] Grupo de Biotecnología y Bioprocesos Marinos, Instituto de Investigaciones Marinas (IIM-CSIC), C/Eduardo Cabello, 6, CP 36208, Vigo, Galicia – España; jvazquez@iim.csic.es (J.A.V.); araceli@iim.csic.es (A.M.); marga@iim.csic.es (M.N.); anais@iim.csic.es (A.I.D.); nsanz@iim.csic.es (N.S.); jvalcarcel@iim.csic.es (J.V.)

[2] Laboratorio de Reciclado y Valorización de Materiales Residuales (REVAL), Instituto de Investigaciones Marinas (IIM-CSIC), C/Eduardo Cabello, 6, CP 36208, Vigo, Galicia – España

[3] Laboratorio de Bioquímica de Alimentos, Instituto de Investigaciones Marinas (IIM-CSIC), C/Eduardo Cabello, 6, CP 36208, Vigo, Galicia – España

* Correspondence: jvazquez@iim.csic.es; Tel.: +34-986-231930 (J.A.V.)

Supplementary Material

B

A

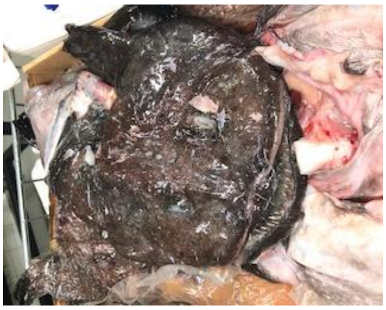

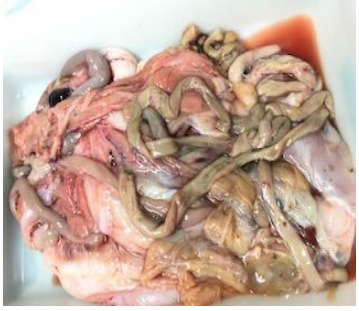

C

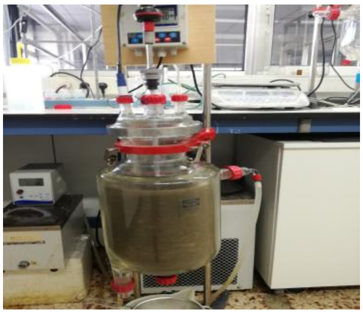

D

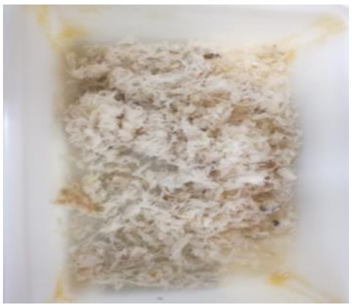

E

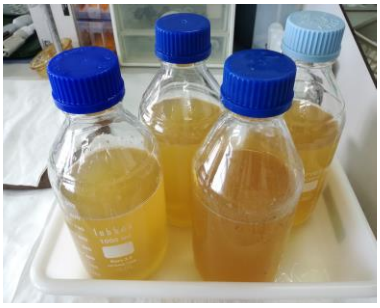

F

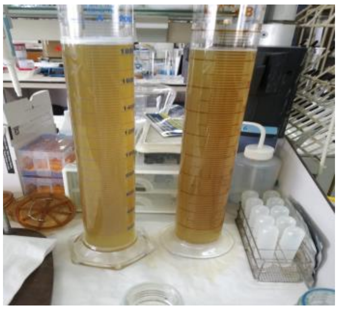

Figure 1. Pictures of monkfish substrates and hydrolysates: A) heads, B) viscera, C) Alcalase hydrolysis of monkfish wastes in a 5L-pH-stat reactor, D) clean bones recovered, E) head liquid FPH, and F) viscera liquid FPH.

Table 1. Experimental domain and coding of the independent variables in the factorial design executed to study the joint effect of pH and temperature on the Alcalase hydrolysis of monkfish heads.

	**Natural values**	
**Coded values**	**pH**	**T (ºC)**
−1.41	6.0	30.0
−1	6.6	37.3
0	8.0	55.0
+1	9.4	72.7
+1.41	10.0	80.0

Codification: $V_c = (V_n - V_0)/\Delta V_n$
Decodification: $V_n = V_0 + (\Delta V_n \times V_c)$
V_n = natural value of the variable to codify
ΔV_n = increment of V_n for unit of V_c
V_0 = natural value in the centre of the domain
V_c = codified value of the variable

Constant conditions
Agitation = 200 rpm
r (S:L) = 1:1
[Alcalase] = 0.2% (*v*/*w*) or 4.8 AU/kg of heads
time of hydrolysis = 3 h
